# Additive Intralesional Interleukin-2 Improves Progression-Free Survival in a Distinct Subgroup of Melanoma Patients with Prior Progression under Immunotherapy

**DOI:** 10.3390/cancers14030540

**Published:** 2022-01-21

**Authors:** David Rafei-Shamsabadi, Saskia Lehr, Max Behrens, Frank Meiss

**Affiliations:** 1 Department of Dermatology and Venereology, Medical Center—University of Freiburg, Faculty of Medicine, University of Freiburg, Hauptstrasse 7, 79104 Freiburg, Germany; david.rafei-shamsabadi@uniklinik-freiburg.de (D.R.-S.); saskia.lehr@uniklinik-freiburg.de (S.L.); 2 Institute of Medical Biometry and Statistics, Faculty of Medicine and Medical Center—University of Freiburg, Zinkmattenstr. 6a, 79108 Freiburg, Germany; behrens@imbi.uni-freiburg.de

**Keywords:** metastatic melanoma, Interleukin-2, intralesional, combination therapy, PD-1 inhibitor

## Abstract

**Simple Summary:**

Despite the immense progress in systemic treatment of advanced unresectable melanoma with immunotherapies, there is still an unmet need for patients showing primary resistance. We show that addition of intratumoral injections of the immune modulator Interleukin-2 can overcome primary resistance in a distinct subset of patients and this effect is associated with an intratumoral increase of certain inflammatory cells supporting the paradigm of turning a “cold” into a “hot” tumor.

**Abstract:**

A considerable amount of melanoma patients show primary resistance to PD-1 and CTLA-4 inhibitors. We have previously reported a beneficial role of intralesional Interleukin-2 (IL-2) in 9 melanoma patients developing new locoregional metastases under immunotherapy. We have now expanded this retrospective cohort to 27 patients. Patients were evaluated for their tumor characteristics, treatment response and progression-free and overall survival (PFS/OS). In 16 patients, tumor biopsies before and under IL-2 treatment were evaluated for immune markers. The median follow-up time was 16 (1–59) months from start of IL-2 treatment. Treatment response of locoregional metastases was seen in 74% of all patients and response of distant organ metastases in 37% of stage IV patients, respectively. A prolonged PFS and OS was significantly associated with absence of active distant metastases (*p* = 0.008), response of locoregional metastases (*p* = 0.002), increase of absolute eosinophil count (AEC) (*p* < 0.001) and an influx of CD8^+^ tumor infiltrating lymphocytes (TILs) (*p* = 0.003). Additional intralesional treatment with IL-2 in patients with locoregional progression under immunotherapy is a well-tolerated, easily feasible therapeutic option especially in patients lacking active distant metastases. A careful patient selection can lead to an improved PFS and OS.

## 1. Introduction

Systemic immunotherapies with Programmed Cell Death Protein 1 (PD-1) and Cytotoxic T-Lymphocyte-Associated Protein 4 (CTLA-4) inhibitors have improved the progression-free survival (PFS) and overall survival (OS) of patients with metastasized melanoma [1]. Nevertheless, at least half of these patients do not respond to these treatments, showing primary resistance to immunotherapy [2]. Recently, it has been shown that patients developing locoregional metastases especially on the lower extremities seem to have lower response rates to PD-1 inhibitors suggesting a biologically diverse behavior [3]. Accessible metastases of the skin and/or the locoregional lymph nodes have been treated intralesionally with oncolytic herpes simplex virus (Talimogene Laherparepvec (TVEC)), Interleukin-2 (IL-2) or IL-2 related agents, showing promising response rates [4,5,6,7,8]. For patients showing locoregional progression under standard systemic PD-1-inhibitor therapy in particular, there is a need for an effective second line treatment. Studies investigating the effect of additive intralesional therapies on a PD-1-inhibitor backbone have been conducted and seem to support the beneficial role of these combinations [9,10]. We have previously described a retrospective cohort of 9 patients showing progressive disease with new locoregional metastases under standard PD-1-inhibitor treatment who received additional intralesional IL-2 as an individual treatment decision [11]. The majority of patients developed a locoregional response to IL-2 treatment, which was accompanied by an influx of CD8^+^ TILs and an increase of the absolute eosinophil count (AEC) in the blood [11]. We have now expanded this cohort to 27 patients. All of them developed locoregional progression on previous PD-1-inhibitor treatment (adjuvant or palliative) and received additional intratumoral IL-2 treatment. We were able to show significant differences in the median PFS and OS for certain subgroups of our cohort. Based on the results of this extended cohort we identified a distinct subset of patients that will benefit most from additional intratumoral IL-2 treatment.

## 2. Materials and Methods

### 2.1. Patients

All 27 patients included in this retrospective study had histologically confirmed stage IV or unresectable stage III melanoma (AJCC 2017) and showed progressive disease (PD) under immunotherapy with PD-1-inhibitors by occurrence of new locoregional metastases. Patients had to display at least one injectable metastasis. As an individual treatment decision, patients received additional intralesional injections with IL-2 while immunotherapy continued. Tumor evaluation was carried out clinically and with ultrasound at all patients’ visits. Radiological tumor assessments were performed every 3 months, pursuant RECIST version 1.1. [12]. Routine laboratory examinations (complete blood count (CBC), lactate dehydrogenase (LDH) and S100) were available for most of the patients at the beginning of combination therapy and at later time points.

### 2.2. Interleukin-2 Treatment

Dilution of IL-2 (PROLEUKIN^®^ S, Novartis Pharma, Basel, Switzerland) in 5% glucose solution to a final concentration of 3 MIU/mL was prepared by the central hospital pharmacy of the Medical Center—University of Freiburg on the day of treatment.

In one treatment session, a maximum of 18 MIU (6 mL) per patient was injected. The volume injected per single cutaneous or subcutaneous metastasis depended on the size as recommended by Weide et al. [4]. The volume injected into nodal metastases did not exceed 0.5 mL (1.5 MIU). A total of 22 patients (81%) received injections into locoregional cutaneous metastases, 15 patients (55.6%) into locoregional subcutaneous metastases, 2 patients (7.4%) into distant cutaneous metastases and 2 patients (7.4%) into distant subcutaneous metastases. In addition, in 7 patients (26%) locoregional nodal metastases were treated with ultrasound guided intranodal injections. The most common side effects were pyrexia, chills and local inflammation (Table S1).

### 2.3. Histology and Immunohistochemistry

Skin biopsies were fixed with formalin, embedded into paraffin and cut into serial sections before staining with hematoxylin and eosin for routine diagnostic. After deparaffinization and heat induced antigen retrieval with EDTA pH9 or citrate pH6 retrieval buffer, immunohistochemical (IHC) staining was performed.

Staining sections were incubated at 4 °C overnight respectively with the following antibodies: Programmed Cell Death 1 Ligand 1 (PD-L1) with a rabbit monoclonal antibody (Cell Signaling Technology; clone: E1L3N; catalog number: #13684; dilution of 1:100) and PD-1 staining with a polyclonal goat antibody (R&D systems; Catalog Number: AF1086; dilution 1:100). Additional antibodies (Ab) for IHC included anti-CD4 Ab (Zytomed, catalog number: BRB042, undiluted) and anti-CD8 Ab (DAKO^TM^, catalog number: M7103, dilution 1:50). Sections incubated with secondary antibody only were used as staining controls. Sections were analyzed using the Dako REAL™ detection system, Alkaline Phosphatase/RED, Rabbit/Mouse (Dako, catalog number: K5005). Pictures of stainings were taken with a Zeiss microscope (Axioscope). For visualization, the program AxioVision (Zeiss, SE64 release 4.9) was used.

For the IHC markers PD-1 and PD-L1 a histology score (H-score) was computed as a semi quantitative approach with the formula: H-Score = (1 × (% cells 1+) + 2 × (% cells 2+) + 3 × (% cells 3+)). Membrane staining intensity (0 = no staining, 1+ = weak staining, 2+ = moderate staining, or 3+ = strong staining [13]).

Using the imaging software QuPath [14], absolute cell numbers per mm^2^ of CD4^+^ and CD8^+^ cells were determined. The positive cell detection and counting tool was used to calculate average cell numbers of at least three diverging tumor sites (magnification ×10).

### 2.4. Statistical Analysis

GraphPad Prism version 5.03 for Windows (GraphPad Software, La Jolla, CL, USA,) was used for statistical analysis and to generate vector graphics. Groups were compared using two-tailed, non-parametric Mann–Whitney test with statistical significance noted as *p* < 0.05.

Survival analyses were conducted using the Kaplan–Meier method. A log-rank test was used to compare survival distributions. Survival was calculated from start of combination therapy to date of progressive disease or melanoma-specific death, respectively, and was censored on the last date the patient was reported to be alive. The *p*-values and 95% confidence intervals were calculated and reported.

Survival analyses and plotting were conducted using IBM Corp. Released 2020. IBM SPSS Statistics for Windows, Version 28.0. IBM Corp: Armonk, NY, USA.

## 3. Results

### 3.1. Clinical Characterization of Patients

In total, 27 patients (56% male and 44% female) with stage III (41%) and IV (59%) melanoma who showed locoregional progression under previous immunotherapy with PD-1-inhibitors were included in this retrospective study as depicted in Table 1 and Table S1. The median age was 73 (22–88). ECOG levels were 0 for 78% and 1 for 22% of patients. In 48% of cases baseline LDH was normal compared to 30% with elevated LDH. In 6 patients or 22% baseline LDH was not available. A total of 14 patients (52%) presented with normal baseline S100 levels and 5 (19%) with elevated levels and in 8 patients (29%) the S100 was not available at baseline. The neutrophil-to-lymphocyte ratio (NLR) was available for 24 patients, 12 of them showed a value above 2.5 and another 12 patients had levels below this cut-off.

A targetable *BRAF*-V600-Mutation was detected in 5 (19%) patients. The median number of locoregional metastases at baseline was 10 (3–100). A total of 9 patients (33%) showed locoregional progression on adjuvant therapy and 18 patients (67%) on palliative therapy. Different types of melanoma were found among patients as follows: 13 (48%) patients with classic cutaneous melanoma, 11 (40%) patients with acral lentiginous melanoma, 1 patient (4%) with uveal melanoma, 1 patient (4%) with mucosal melanoma and 1 patient (4%) with a cancer of unknown primary (CUP). The median follow-up time for all patients was 16 (1–59) months.

### 3.2. Intralesional IL-2 Treatment Leads to Locoregional Response in Most Patients and Can Induce Abscopal Effects in Distant Metastases

All 27 patients included in this study presented with injectable locoregional cutaneous and subcutaneous metastases and some with additional lymph node metastases. Of these patients, 11 (41%) showed a complete response of locoregional metastases under IL-2 treatment and 9 (33%) had a partial response (please refer to Table 2). Another 7 (26%) patients developed progression of locoregional metastases despite IL-2 injections. From the 16 patients presented with stage IV melanoma 1 (6%) had a complete response of a distant lung metastasis, 5 (31%) showed a partial response, 1 (6%) a stable disease and 7 (44%) developed a progression of their distant metastases. In 2 (13%) patients, assessment of response in distant metastases was not applicable due to successful prior treatment leading to an inactive status or due to fast progression leading to death and making the first staging impossible. In summary, the majority of patients showed response of locoregional metastases to IL-2 treatment. In those patients with response of non-injected distant metastases (37%) under IL-2 we presume a reaction pattern which is referred to as an abscopal effect in the literature [15,16].

### 3.3. Lack of Active Distant Metastases and Locoregional Response to IL-2 Is Associated with Prolonged Progression-Free and Overall Survival

**Figure 1:**
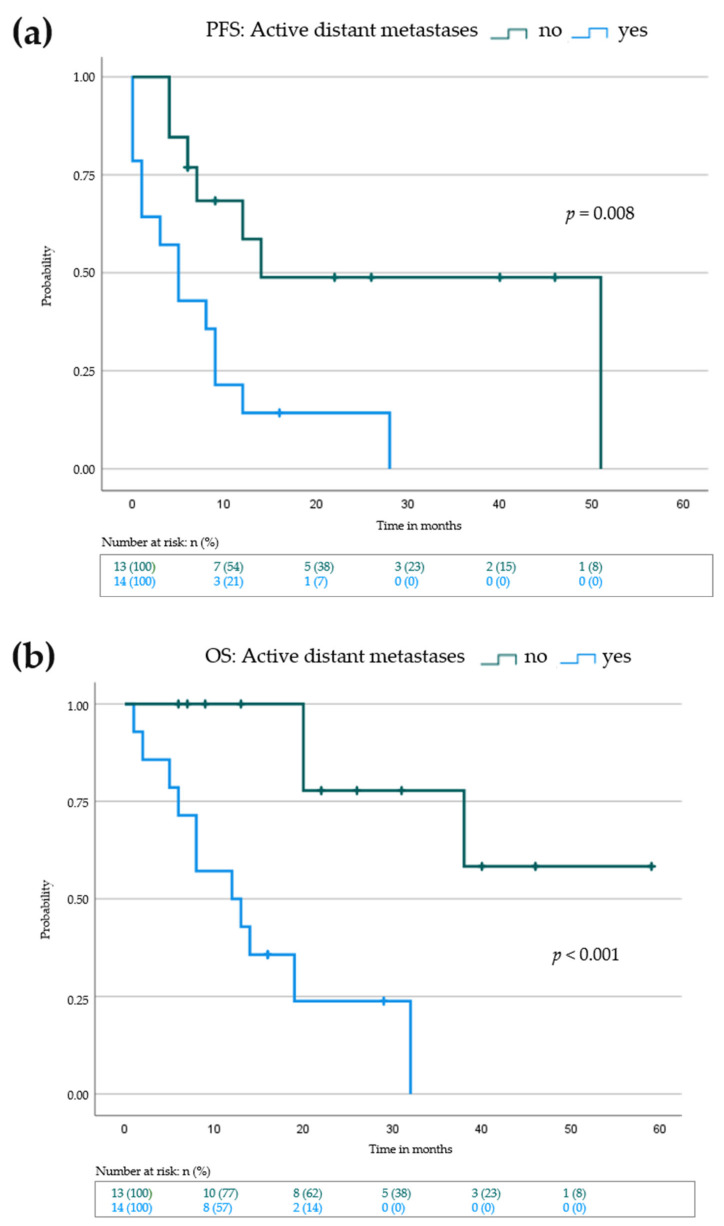
Kaplan–Meier survival blot showing progression-free survival (PFS) in (**a**) and overall survival (OS) in (**b**) from beginning of IL-2 treatment for patients with active distant metastasis (blue curve) vs patients without active distant metastasis (green curve). Vertical dashes within the curves indicate censored patients. *p*-value indicates statistical significance of the log rank test.

**Figure 2:**
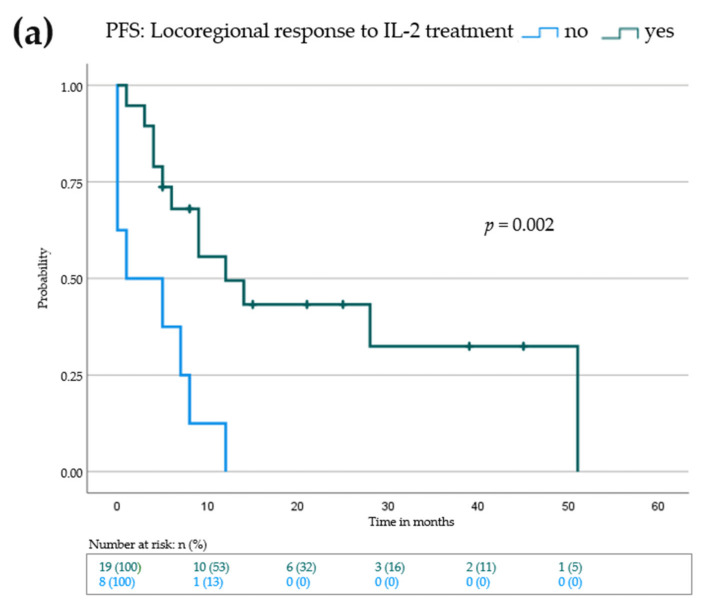

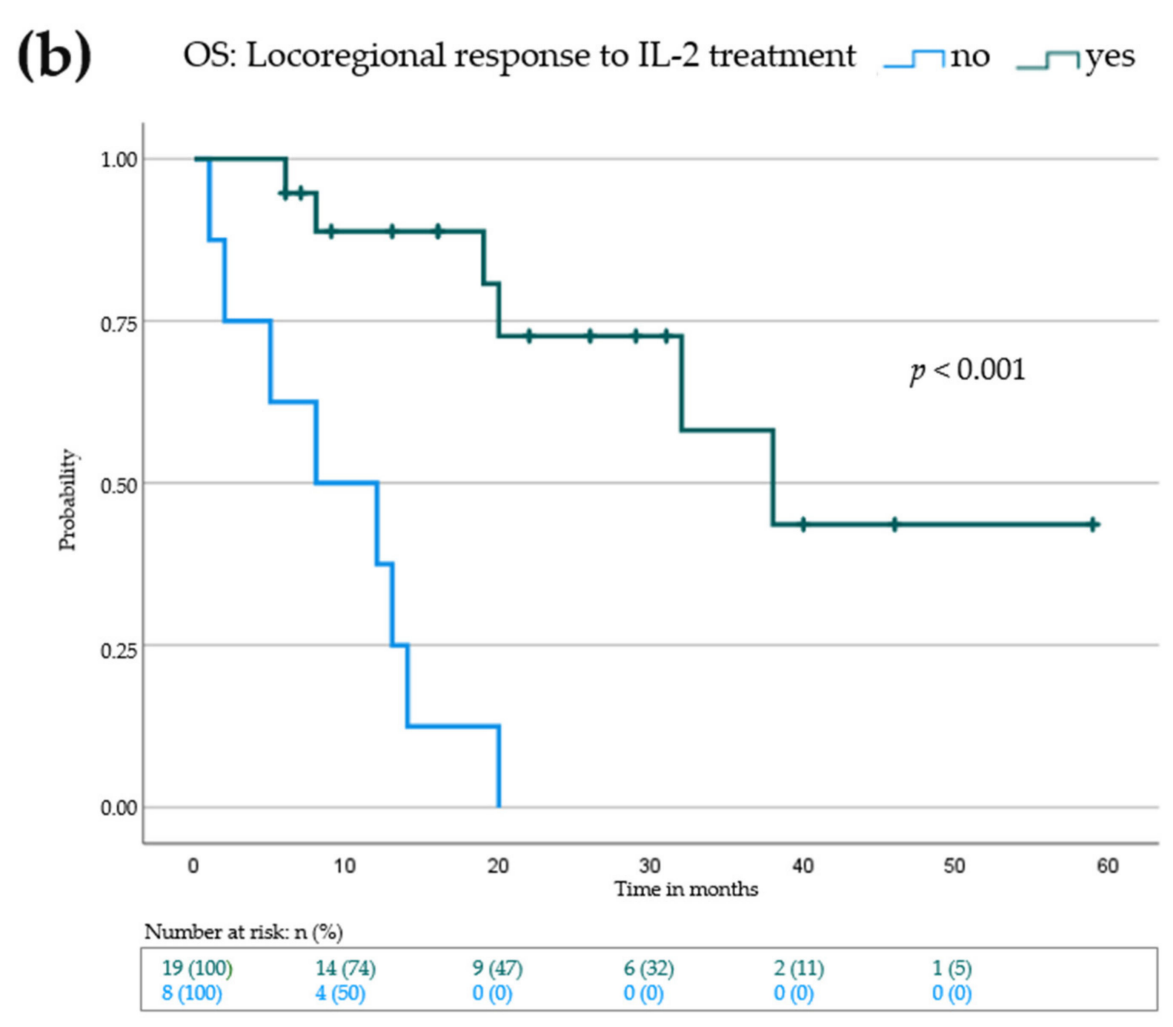
Kaplan–Meier survival blot showing progression-free survival (PFS) in (**a**) and overall survival (OS) in (**b**) from beginning of IL-2 treatment for patients with locoregional response to IL-2 treatment (green curve) vs no locoregional response to IL-2 treatment (blue curve). Vertical dashes within the curves indicate censored patients. *p*-value indicates statistical significance of the log rank test.

Progression-free survival (PFS) is the benchmark analysis for the efficacy of an oncological drug since it describes the time to next treatment, if needed. In addition, we determined the melanoma specific overall survival (OS) for our patients. We defined the PFS for all 27 patients as the time in months from the beginning of additive IL-2 treatment until progression of locoregional and/or distant metastases or until the time point of the last visit if no progression occurred. Melanoma specific OS data shared the same starting point as PFS and lasted until death due to melanoma progression or until the time point of the last visit if no death occurred. The median PFS for all patients was 8 (0–51) months; the median OS was 16 (2–59) months. We performed Kaplan–Meier analysis with a log rank test to investigate possible differences in baseline characteristics that may have an influence on the PFS or OS (Figure 1, Table 3 and Table S2). Neither sex nor age over 70 at baseline had a significant effect on PFS (9 months (female) vs. 7 months (male), *p* = 0.468) or OS (12 months (age <70) vs. 7 months (age >70), *p* = 0.613) (Table 3 and Table S2). There were differences in the median PFS and OS of patients who progressed on adjuvant (14 months PFS and 32 months OS) vs. palliative (6 months PFS and 14 months OS) therapy, however this did not reach statistical significance (Table 3 and Table S2). As expected, patients with active distant metastases had a significantly lower median PFS than patients with stage III melanoma or pretreated, inactive distant metastases (median PFS: 5 vs. 14 months, *p* = 0.008) (Figure 1a and Table 3). The effect was even more pronounced when looking at the OS (Figure 1b and Table S2). Here, the median OS for patients lacking active distant metastases was not reached (median OS: 12 months vs. not reached, *p* < 0.001). Interestingly, no differences in PFS or OS were seen for baseline LDH levels (9 months (normal LDH) vs. 9 months (elevated LDH), *p* = 0.716), or *BRAF*-V600-Mutation status (8 months (wild type) vs. 14 months (mutated), *p* = 0.717). We further evaluated baseline S100 levels and the NLR. Again, no significant differences in PFS or OS could be detected (Table 3 and Table S2). Patients who showed a response of their locoregional metastases to IL-2 treatment had a significantly higher median PFS (1 month (no response) vs. 12 months (response), *p* = 0.002) and OS (8 months (no response) vs. 38 months (response), *p* < 0.001), respectively (Figure 2, Table 4 and Table S3). Thus, patients without active distant metastases and locoregional response may benefit the most from additional IL-2 treatment.

### 3.4. Elevation of Eosinophils in the Peripheral Blood and Increase of CD8^+^ Tumor Infiltrating Lymphocytes Is Associated with Improved Progression-Free and Overall Survival

**Figure 3:**
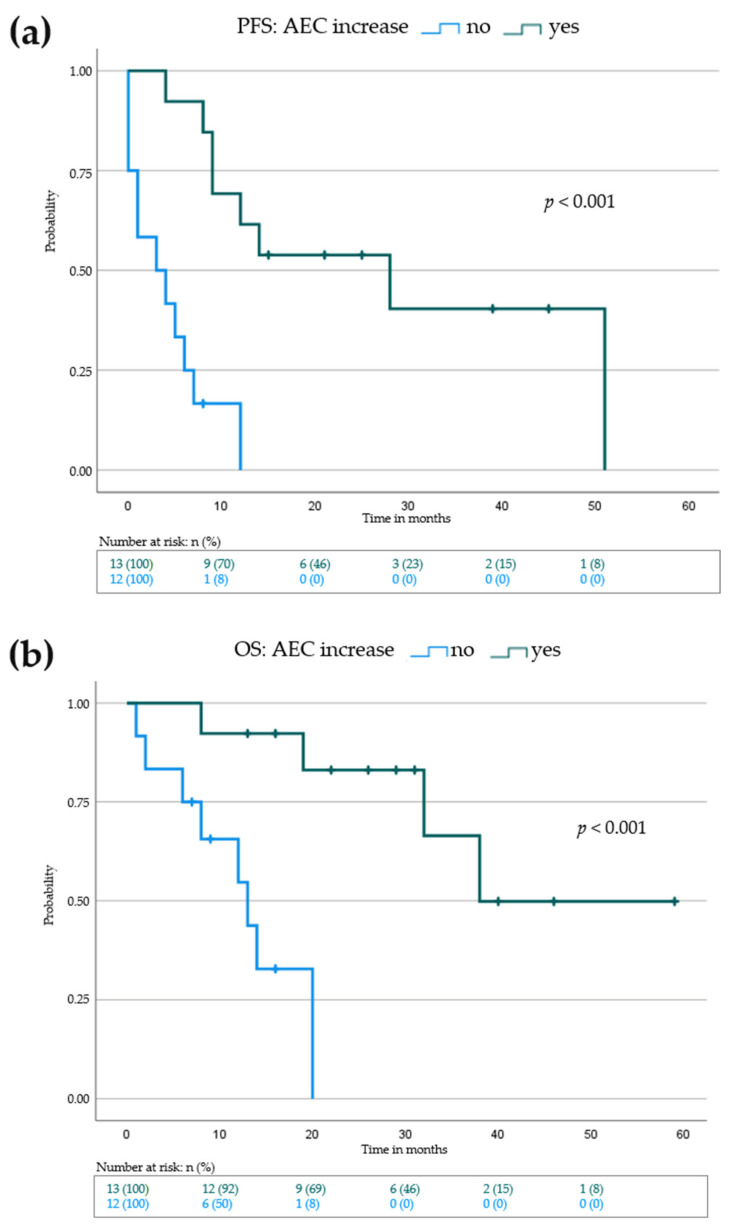
Kaplan–Meier survival blot showing progression-free survival (PFS) in (**a**) and overall survival (OS) in (**b**) from beginning of IL-2 treatment for patients with increase of absolute eosinophil count (green curve) in the peripheral blood vs patients without increase of absolute eosinophil count in the peripheral blood (blue curve). Vertical dashes within the curves indicate censored patients. *p*-value indicates statistical significance of the log rank test.

Next, we sought to investigate possible biological markers occurring under IL-2 treatment that may be associated with PFS and OS outcome in our cohort. Increase of absolute eosinophil count (AEC) in peripheral blood during IL-2 treatment correlated with a significantly prolonged PFS (3 months (no AEC increase) vs. 28 months (AEC increase), *p* = <0.001) and OS (13 months (no AEC increase) vs. 38 months (AEC increase), *p* = <0.001) (Figure 3, Table 4 and Table S3). Thus, our data suggest that the increase of AEC in the peripheral blood during IL-2 treatment as a surrogate marker for an improved PFS and OS.

From 16 patients in our cohort tumor material, i.e., skin metastases, was available before start of IL-2 treatment and under intralesional therapy. A total of 4 of these patients had a progression of their locoregional metastases despite IL-2 treatment and the remaining 12 patients developed at least a partial (*n* = 3) or a complete response (*n* = 9).

We performed immunohistochemical staining of tumor material to detect CD4^+^ and CD8^+^ tumor infiltrating lymphocytes (TILs) as well as immunosuppressive markers such as PD-1 and PD-L1. CD4^+^ and CD8^+^ T cells (absolute numbers of per mm^2^) within the tumor microenvironment were measured using a software based quantitative approach. PD-1 and PD-L1 staining was assessed semi quantitatively using the H-Score (see methods section).

**Figure 4:**
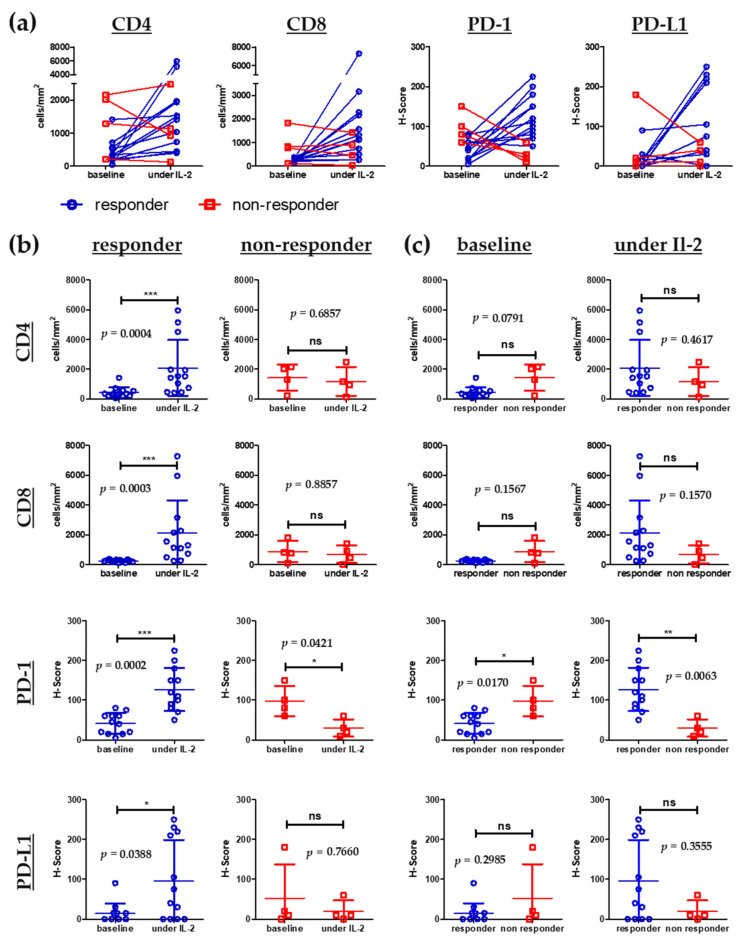
Immunohistochemistry staining of melanoma metastases at baseline (before start of IL-2 treatment) and under IL-2 treatment. (**a**) Graphs display intraindividual changes in cell numbers of CD4^+^ and CD8^+^ tumor infiltrating lymphocytes (TILs) as well as staining intensities for Programmed Cell Death Protein 1 (PD-1) and Programmed Cell Death 1 Ligand 1 (PD-L1) in tumor biopsies from patients with response of locoregional metastasis (responders) compared to biopsies from patients without response of locoregional metastasis (non responders). (**b**) Characterization of differences of the inflammatory infiltrate between biopsies taken at baseline and under IL-2 treatment for responders and non responders. (**c**) Characterization of differences of the inflammatory infiltrate between biopsies from responders and non responders at baseline and under IL-2 treatment. Absolute numbers per mm^2^ are displayed for CD4^+^ and CD8^+^ positive cells. Staining intensities of PD-1 and PD-L1 were quantified using the H-score (for detailed calculation see methods section). Patients responding to combination therapy are displayed in blue, non-responding patients are shown in red. *n* = 16 taken biopsies. Results are shown as mean ± SD. Two-tailed, non-parametric Mann–Whitney test was used to compare groups. Statistical significance was noted as * *p* < 0.05, ** *p* < 0.01, *** *p* < 0.001. ns = not significant.

In the group of patients showing locoregional response to IL-2 therapy, an intraindividual increase of CD8^+^ and CD4^+^ as well as PD-1^+^ T cells under IL-2 therapy in most of the patients was evident when compared to baseline cell numbers (Figure 4a). T-test analysis of mean cell numbers at baseline compared to specimens under IL-2 therapy showed a highly significant increase in CD4^+^ and CD8^+^ T cells in the group of responders (Figure 4b). A similar trend could be detected for the PD-L1 staining however to a lesser extent (Figure 4a,b). In contrary, in metastases from the 4 patients showing progression of locoregional metastases under IL-2 therapy we could not detect an increase of CD8^+^ TILs, and in some specimens even a decrease of CD8^+^ TILs occurred (Figure 4a). CD4^+^ T cells also showed no increasing trend except for one patient (Figure 4a). PD-1 staining clearly decreased under IL-2 treatment whereas PD-L1 staining showed no clear trend (Figure 4a). The decrease in PD-1^+^ T cells was significant in the non-responding group (Figure 4b). Finally, when comparing responders and non-responders at baseline and under IL-2 therapy, there was an inverse relation of PD-1^+^ T cell numbers, i.e., PD-1 staining was significantly higher at baseline in the non-responder group and this relation reversed under IL-2 therapy (Figure 4c).

**Figure 5:**
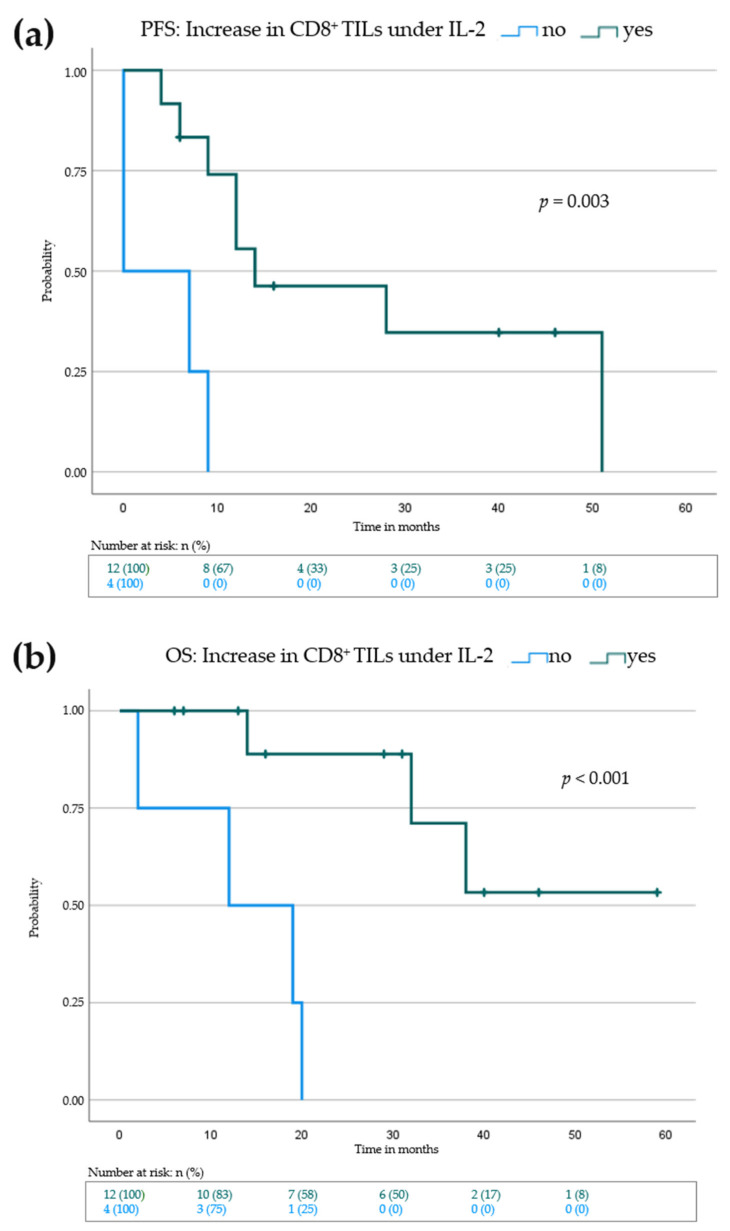
Kaplan–Meier survival blot showing progression-free survival (PFS) in (**a**) and overall survival (OS) in (**b**) for patients with increase of CD8^+^ tumor infiltrating lymphocytes (green curve) vs patients with no increase of CD8^+^ tumor infiltrating lymphocytes (blue curve). Vertical dashes within the curves indicate censored patients. *p*-value indicates statistical significance of the log rank test.

Next, we investigated if an increase of the above-mentioned immune markers in melanoma metastases during IL-2 treatment may have a correlation with PFS and OS. The median PFS was higher in patients showing an increase of CD4^+^ (PFS: 7 months (no increase) vs. 14 months (increase)), CD8^+^ (0 months (no increase) vs. 14 months (increase)) or PD-1^+^ (7 months (no increase) vs. 12 months (increase)) TILs. However, when performing the log rank test only the increase of CD8^+^ T cells showed a highly significant difference (*p* = 0.003) (Figure 5a, Table 4 and Table S3). Increase in PD-L1 staining did not correlate with mean PFS (Table 4). When looking at the OS, an increase in CD8^+^ and CD4^+^ T cells was associated with a prolonged OS (CD4^+^: 19 months (no increase) vs. median not reached (increase), *p* = 0.004), CD8^+^: 12 months (no increase) vs. median not reached (increase), *p* < 0.001) (Figure 5b and Figure S1, Table S3).

Thus, increase of especially CD8^+^ TILs can be detected in patients showing locoregional response to IL-2 treatment and is associated with prolonged PFS and OS. Due to the small sample number in this analysis, the results should be seen as a trend, that needs to be verified in a large scale cohort.

### 3.5. Influx of CD8^+^ T Cells into Melanoma Metastases Correlates with Clinical Response

**Figure 6:**
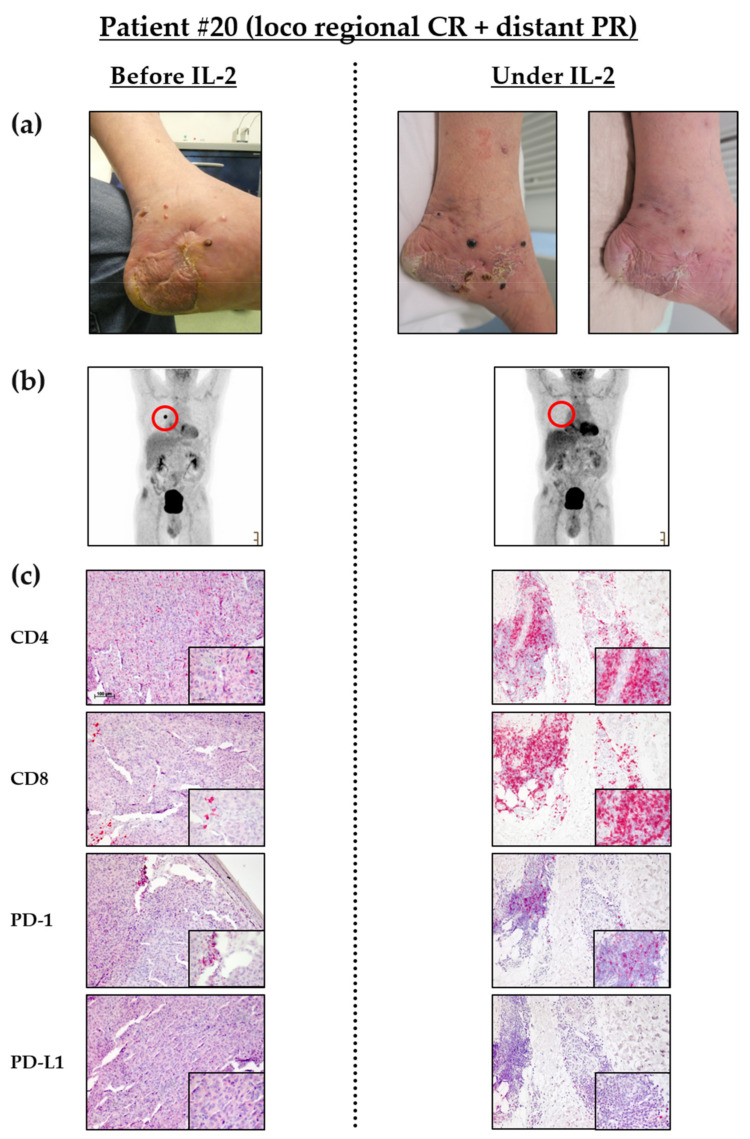
Clinical course of patient 20: (**a**) Representative clinical pictures of cutaneous metastases of the left malleolus medialis at different time points. i.e., before (left picture) and under IL-2 treatment (right pictures: after 5 (left) and 14 (right) received injections, 30 and 102 MIU, respectively, in total). (**b**) PET-CT scans before and under IL-2 treatment displaying a pulmonary metastasis (red circle) that shows response to combined therapy. (**c**) Representative immunohistochemistry staining of melanoma metastases. Biopsies were taken before and under treatment with IL-2. Scale bar = 100 μm; Insert: scale bar = 50 μm; CR = complete response; PR = partial response.

As depicted in Figure 6, one patient presented with satellite and in-transit metastases at his left foot. He experienced a good clinical response of these metastases under IL-2 treatment (Figure 6a). In parallel, his lung metastases, which were present before the start of IL-2, nearly completely regressed under additional IL-2 treatment (Figure 6b). When comparing skin metastases in this patient from baseline to being under IL-2 treatment, a marked increase of tumor infiltrating CD4^+^, CD8^+^ and PD-1^+^ T cells is seen (Figure 6c). Under IL-2, these cells clearly show a more tumor infiltrative behavior compared to baseline. A comparable scenario was observed for another patient who developed a strong inflammatory response of his skin metastases to IL-2 leading to a complete response (Figure S2). A third patient on the other hand showed no benefit from IL-2 treatment leading to quickly-progressing skin metastases (Figure S3a). No influx of CD8^+^ TILs, but a strong influx of CD4^+^ T cells was seen. The latter was most likely attributed to an underlying chronic lymphocytic leukemia (CLL) (Figure S3b). Later, the patient experienced further progression with distant metastases and died from his melanoma diagnosis. These patient cases further support the observation that clinical response especially in locoregional metastases is related to an increased influx of CD8^+^ TILs and may even induce abscopal effects in distant metastases.

## 4. Discussion

The fight to overcome primary resistance to PD-1-inhibitor therapy in melanoma patients is of major clinical and scientific interest. Long et al., have investigated 355 patients in a multicenter, retrospective, cohort study with metastatic melanoma resistant to anti-PD-(L)1 (nivolumab, pembrolizumab or atezolizumab) that received either ipilimumab monotherapy or ipilimumab plus anti-PD-1 therapy [2]. Ipilimumab plus anti-PD-1 showed better efficacy than ipilimumab alone with a higher objective response rate (31%), longer median progression-free (3.0 months), and longer overall survival (20.4 months), with a similar rate of grade 3–5 toxicities [2]. This quite small median PFS difference however also highlights that most patients with a primary resistance to anti PD-1-monotherapy show an early progression even when escalating to combination immune checkpoint inhibitor therapy. The question is whether decision for second line therapy in patients showing primary resistance to immunotherapy can be supported by certain individual factors which predict a good treatment response, i.e., personalized oncological therapy.

Not all patients that progress under PD-1-inhibitor therapy are suitable for therapy escalation to combined immunotherapy with ipilimumab plus anti-PD-1 due to older age, underlying comorbidities or their refusal to receive this therapy escalation. In case of progression patterns restricted to or being mainly characterized by locoregional metastases accessible for intralesional therapy, combinational therapeutic approaches of additional intralesional treatments and systemic immunotherapies should be considered, especially in patients lacking targetable mutations (e.g., BRAF-V600) [17]. Therapeutic use of intralesional agents in combination with checkpoint inhibitors is an emerging field. In our current retrospective study, we provide further evidence for the successful treatment of PD-1-inhibitors in combination with intralesional IL-2. 

The possible relevance of baseline biomarkers in the blood and the tumor microenvironment for pre-treatment selection and prognosis in melanoma patients has been widely discussed in the literature [18,19]. We could not detect significant differences in the expression of baseline or follow-up biomarkers in the tumor microenvironment between responding and non-responding patients except for PD-1 (Figure 4c). Non-responding patients presented with higher baseline expression of PD-1 than responders and this relation completely reversed under IL-2 treatment. As depicted in Figure 4b, responding patients clearly show an increase of CD4, CD8, PD-1 and PD-L1 expression under IL-2 therapy. Thus, our study supports the hypothesis of Ribas et al. [9] who demonstrated that dynamic changes in the TME are more relevant than baseline levels. Responses to combination of anti-PD-1 and TVEC appeared independent of baseline CD8^+^ TIL infiltration but CD8^+^ influx was present in most responders [9]. Thus, the dynamic change in the tumor microenvironment seems to be a good predictor for treatment response to intralesional tumor therapies. Furthermore, neither baseline LDH, S100 nor the NLR had a significant impact on PFS or OS (Table 3 and Table S2). In accordance with the dynamic change hypothesis, the AEC increase in the blood under combination therapy was a good predictor for prolonged PFS and OS. The most predictive baseline value for a poor outcome was the presence of active distant metastases as already shown in other cohorts [19]. Taken together, we could not identify a reliable predictive baseline factor in the TME or the peripheral blood that might help in pre-treatment patient selection.

In conclusion, in our expanded cohort, we could identify a subgroup of patients that might be most suitable for additional IL-2 treatment as seen by a significant improvement of PFS and OS. These patients are lacking active distant metastases at the start of IL-2, develop a good clinical response of their locoregional metastases accompanied by an increase of AEC in the peripheral blood and show an influx of CD8^+^ TILs in the IL-2-treated metastases.

Increased response rates to intralesional IL-2 monotherapy have been described to be higher in patients with locoregional metastases alone or without visceral metastases [4]. However, none of these patients had received prior immunotherapy with anti-PD-1 or anti-CTLA-4-antibodies and IL-2 was not given in combination with a systemic therapy [4]. Thus, intralesional IL-2 is a drug showing the most benefit in distinct subgroups of melanoma patients and we now provide evidence that this also holds true even after progression on previous immunotherapy.

This study has some limitations. Due to the relatively small number of patients, the statistical analysis is purely descriptive especially when looking at the PFS and OS data gained from immunohistochemical analysis in metastases from 16 patients out of 27. Moreover, our cohort also comprised patients with non-cutaneous melanomas (uveal, mucosal) and one patient with a CUP. To exclude a possible bias in the data by including non-cutaneous melanomas, we performed a Kaplan–Meier analysis for the 24 patient cohort of cutaneous melanomas only. Tables S4 and S5 show that *p*-values for the most relevant baseline and follow up factors do not significantly differ compared to the entire cohort. Time points of skin biopsies and blood samples were not standardized due to the retrospective character of this study. The study does not include a control arm with patients not receiving additional IL-2 but an immediate change of their systemic therapy. However, this is a retrospective cohort and additional IL-2 treatment was based on individual decisions in line with the patients will. Nevertheless, in this expanded cohort we could confirm previous findings such as the correlation of response with AEC and CD8^+^ TIL increase under IL-2. We could now identify a patient subgroup that benefits most from additional IL-2 treatment leading to a more personalized oncological use of therapeutic concept.

It is arguable that intratumoral treatment with IL-2 may have diverse effects on the tumor microenvironment, i.e., not only induction of CD8^+^ cytotoxic T cells or NK cells but also induction of regulatory T cells (Tregs) [20,21]. The latter are supposed to be a key player in an immunosuppressive microenvironment in solid tumors leading to negative effects on systemic or intratumoral therapies [22,23,24]. These pleiotropic effects of IL-2 are mediated through the variable structure of the IL-2 receptor expressed by different immune cells [24]. To overcome this negative effect, a new therapeutic generation of IL-2 containing drugs has been developed [6]. The IL-2 prodrug Bempegaldesleukin for example is an engineered PEGylated IL-2 agonist that preferentially binds to the IL-2-receptor type predominantly expressed on T effector cells and NK cells and to a lesser extent to the IL-2-receptor type expressed on Tregs [24,25,26]. This leads to intratumoral proliferation, activation and effector function of CD8^+^ T and NK cells without expanding intratumoral Tregs implicating less tumor suppressive effects [24,25]. Furthermore, the antibody–cytokine fusion protein Daromun is a combined molecule consisting of IL-2 and TNF fused to the monoclonal antibody L19, which is specific to the alternatively spliced extra-domain B of fibronectin, a marker of tumor angiogenesis [7]. The drug is administered intralesionally and showed high response rates in injected and non-injected lesions [7] leading to the phase III neo-DREAM trial in patients with resectable stage IIIB/C melanoma [27].

In our cohort IL-2 injections were largely well tolerated in combination with systemic anti-PD-1 inhibitor treatment. The most common side effects were pyrexia, chills and local inflammation. Interestingly, patients treated with nodal metastases did not show higher rates of or more severe side effects. Nevertheless, one patient experienced a syncope twice after cutaneous injection, which lead to treatment discontinuation. This was however the only limiting toxicity in our cohort.

In summary, these new developments in intralesional, IL-2-based therapies will further improve the outcome of certain melanoma patients harboring accessible, locoregional metastases. Since these drugs are not yet approved for daily routine use, we focused on the already available, well-tolerated conventional IL-2 for the treatment of our patients and achieved promising results, which should be confirmed in larger prospective cohort studies possibly involving next generation IL-2-based agents.

## 5. Conclusions

Our data indicate that patients lacking active distant metastases and developing an increase of AEC in the blood as well as an influx of CD8^+^ TILs may benefit most from additional intratumoral IL-2 after progression on PD-1-inhibitor treatment.

## Figures and Tables

**Table 1:** Baseline Patient characteristics.

Characteristics	Patients (*n* = 27)
**Median age (years)**	73 (22–88)
**Sex**	
Male	15 (56%)
Female	12 (44%)
**ECOG ^1^**	
0	21 (78%)
1	6 (22%)
**AJCC ^2^ stage (2017)**	
III	11 (41%)
IIIB	2 (7%)
IIIC	9 (34%)
IV	16 (59%)
M1a	3 (11%)
M1b	4 (15%)
M1c	7 (26%)
M1d	2 (7%)
**Baseline LDH ^3^**	
Normal	13 (48%)
Elevated	8 (30%)
NA ^4^	6 (22%)
**Baseline S100**	
Normal	14 (52%)
Elevated	5 (19%)
NA	8 (29%)
**Baseline NLR ^5^**	
≤2.5 (*n* = 12)	12 (44%)
>2.5 (*n* = 12)	12 (44%)
NA	3 (11%)
**BRAF-V600-Mutation**	
Wild type	22 (81%)
Positive	5 (19%)
**Median number of locoregional metastases**	10 (3–100)
**Locoregional Progression on**	
Adjuvant therapy	9 (33%)
Palliative therapy	18 (67%)
**Type/localization of primary melanoma**	
Cutaneous	13 (48%)
Acral lentiginous	11 (40%)
Uveal	1 (4%)
CUP ^6^	1 (4%)
Mucosal	1 (4%)
**Median follow up time, months**	16 (1–59)

^1^ Eastern Cooperative Oncology Group performance status score. ^2^ American Joint Committee on Cancer, Melanoma of the Skin Staging, 8th edition. ^3^ Lactate dehydrogenase. ^4^ Not available. ^5^ Neutrophil-to-lymphocyte ratio. ^6^ Cancer of unknown primary.

**Table 2:** Tumor responses to IL-2 treatment.

Type of Response	Patients
**Locoregional (Stage III and IV) ^1^**	**(*n* = 27)**
Complete response	11 (41%)
Partial response	9 (33%)
Progressive disease	7 (26%)
**Distant organ metastases (Stage IV only) ^1^**	**(*n* = 16)**
Complete response	1 (6%)
Partial response	5 (31%)
Stable disease	1 (6%)
Progressive disease	7 (44%)
NA	2 (13%)

^1^ American Joint Committee on Cancer, Melanoma of the Skin Staging, 8th edition.

**Table 3:** Progression-free survival times (baseline characteristics).

Condition	Median PFS	95% CI ^1^	*p* Values
**Sex**			
Female (*n* = 12)	9	3.0–17.9	0.468
Male (*n* = 15)	7	1.8–12.2	
**Age over 70**			
No (*n* = 12)	12	6.9–17.0	0.613
Yes (*n* = 15)	7	3.5–10.5	
**Locoregional progression on:**			
Adjuvant therapy (*n* = 9)	14	- *	0.125
Palliative therapy (*n* = 18)	6	1.8–10.2	
**Active distant metastases at baseline**			
No (*n* = 13)	14	0.0–33.5	0.008
Yes (*n* = 14)	5	1.4–8.6	
**Baseline LDH ^2^**			
Normal (*n* = 13)	9	0.0–19.6	0.716
Elevated (*n* = 8)	9	2.5–15.5	
**Baseline S100**			
Normal (*n* = 14)	12	2.8–21.2	0.321
Elevated (*n* = 5)	5	0.0–13.6	
**Baseline NLR ^3^**			
≤2.5 (*n* = 12)	9	0.5–17.5	0.706
>2.5 (*n* = 12)	8	2.0–13.9	
***BRAF*-V600-Mutation**			
Wild type (*n* = 22)	8	4.7–11.3	0.717
Positive (5)	14	0.0–29.6	

^1^ 95% confidence interval of median. ^2^ Lactate dehydrogenase. ^3^ Neutrophil-to-lymphocyte ratio. * No CI due to low event numbers. *p*-value indicates statistical significance of the log rank test.

**Table 4:** Progression-free survival times (values under therapy).

Condition	Median PFS	95% CI ^1^	*p* Values
**Increase of AEC ^2^ under IL-2**			
No (*n* = 12)	3	0.0–8.9	<0.001
Yes (*n* = 13)	28	5.9–12.1	
**Locoregional response to IL-2**			
No (*n* = 8)	1	0.0–7.9	0.002
Yes (*n* = 19)	12	2.5–21.5	
**Increase in CD4^+^ TILs ^3^**			
No (*n* = 5)	7	0.0–22.0	0.140
Yes (*n* = 11)	14	9.0–18.9	
**Increase in CD8^+^ TILs**		- *	
No (*n* = 4)	0	0.0–36.6	0.003
Yes (*n* = 12)	14		
**Increase in PD-1 staining ^4^**			
No (*n* = 6)	7	0.0–17.8	0.365
Yes (*n* = 10)	12	7.2–16.7	
**Increase in PD-L1 staining ^5^**			
No (*n* = 9)	12	3.2–20.7	0.669
Yes (*n* = 7)	12	5.5–18.5	

^1^ 95% confidence interval of median ^2^ Absolute eosinophil count. ^3^ Tumor infiltrating lymphocytes. ^4^ Programmed Cell Death Protein 1. ^5^ Programmed Cell Death 1 Ligand 1. * No CI due to low event numbers. *p*-value indicates statistical significance of the log rank test.

## Data Availability

The data used and/or analyzed during the current study are available from the corresponding author on reasonable request.

## Supplementary Materials

The following are available online at https://www.mdpi.com/article/10.3390/cancers14030540/s1, Figure S1: Kaplan–Meier survival blots showing progression-free survival and overall survival for patients with increase and no increase of CD4^+^ tumor infiltrating lymphocytes. Figure S2: Clinical course of patient 27. Figure S3: Clinical course of patient 3. Table S1: Detailed patient characteristics. Table S2: Overall survival times (baseline characteristics). Table S3: Overall survival times (values under therapy). Table S4: Progression-free survival times (Cancer of unknown primary, uveal and mucosal melanoma excluded). Table S5: Overall survival times (Cancer of unknown primary, uveal and mucosal melanoma excluded).
