# Supplementary material for: Additive Intralesional Interleukin-2 Improves Progression-Free Survival in a Distinct Subgroup of Melanoma Patients with Prior Progression under Immunotherapy
Source: Cancers (Basel). 2022 Jan 21;14(3):540. doi: 10.3390/cancers14030540 (PMC8833633; doi:10.3390/cancers14030540)
Supplement: Supplementary file 1 [file cancers-14-00540-s001.zip › cancers-1463269-suppl-final.pdf]

Supplementary Figures

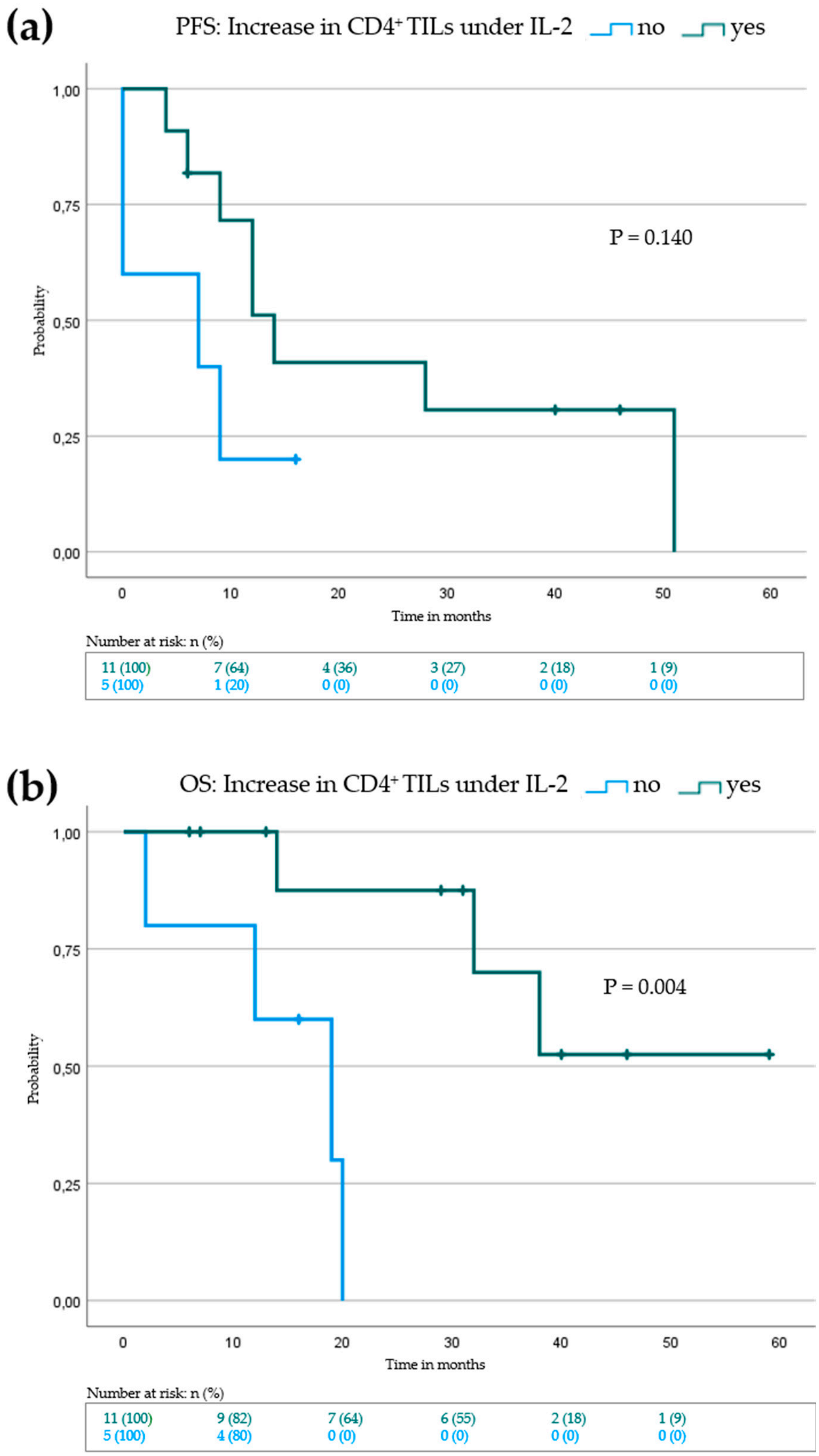

Figure S1: Kaplan-Meier survival blot showing progression free survival (PFS) in **(a)** and overall survival (OS) in **(b)** for patients with increase of CD4⁺ tumor infiltrating lymphocytes (green curve) vs patients with no increase of CD4⁺ tumor infiltrating lymphocytes (blue curve). Vertical dashes within the curves indicate censored patients. *P*-value indicates statistical significance of the log rank test.

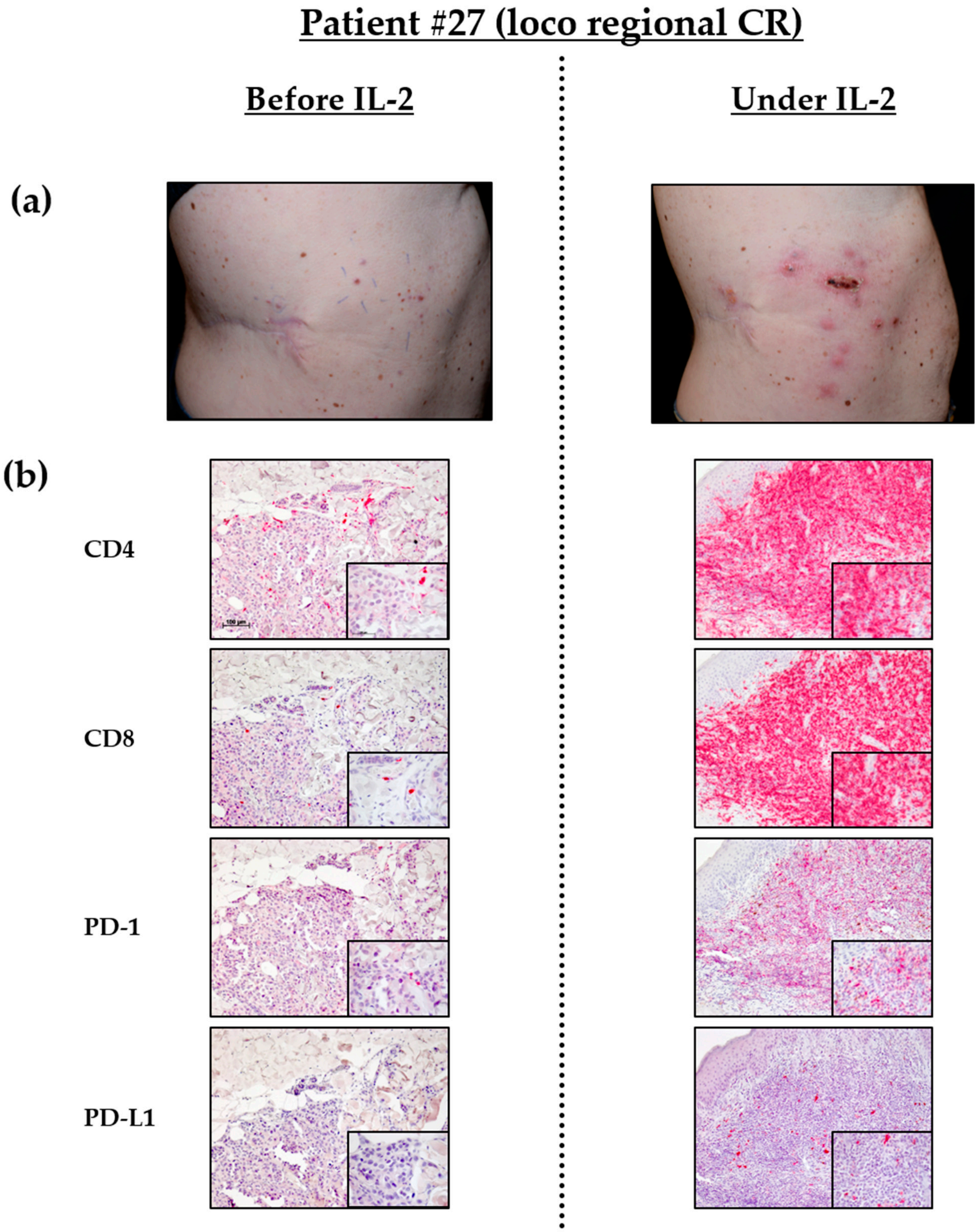

Figure S2: Clinical course of patient 27: **(a)** Representative clinical pictures of cutaneous metastasis of the right flank before (left picture) and under IL-2 treatment (right picture: after 3 injections received, 18 MIU in total). (**b)** Representative immunhistochemistry staining of melanoma metastases. Biopsies were taken before and under treatment with IL-2. Scale bar = 100 µm; Insert: scale bar = 50 µm. CR = complete response.

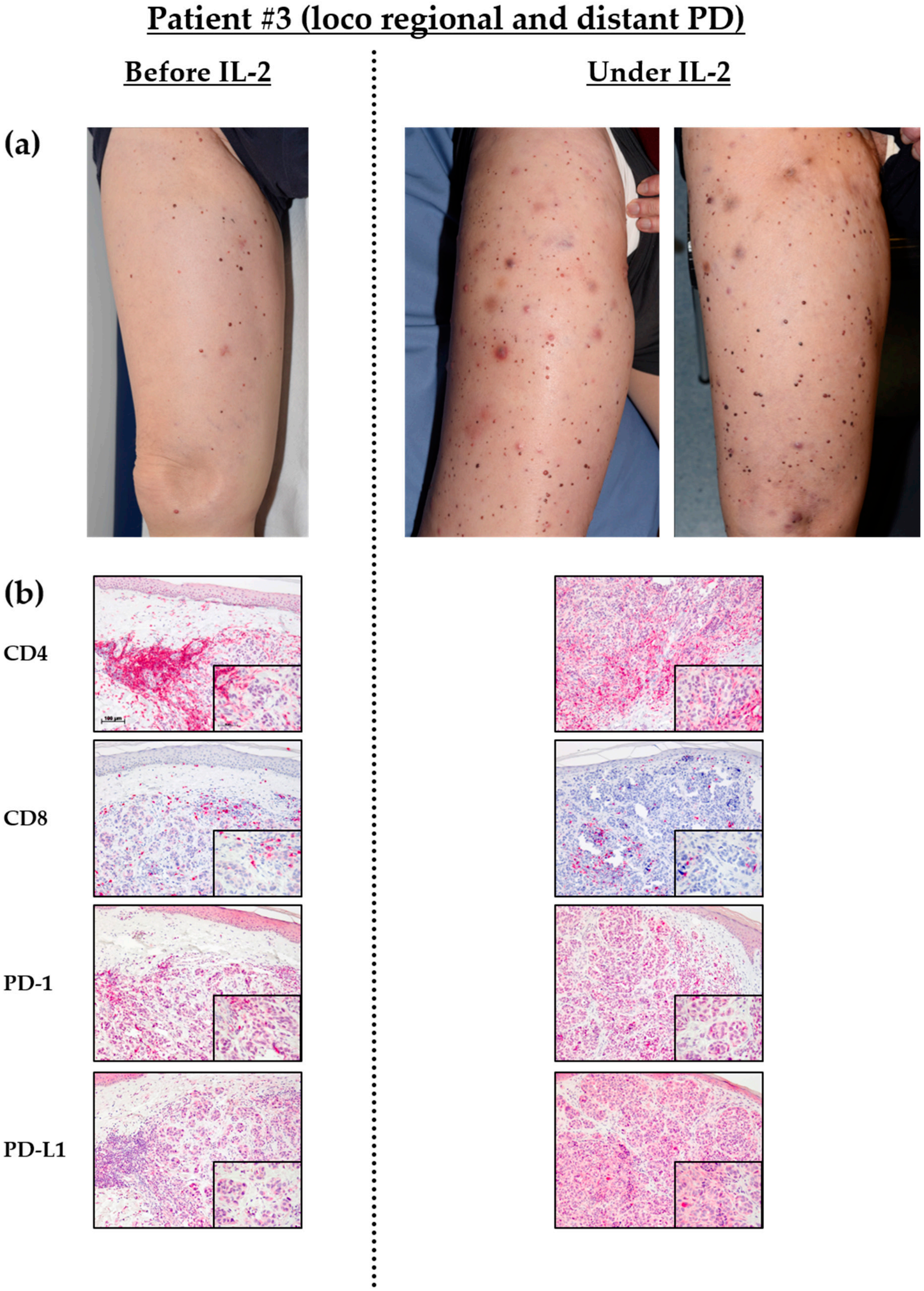

Figure S3: Clinical course of patient 3: (**a**) Representative clinical pictures of cutaneous metastasis of the right upper leg at different time points. i.e. before (left picture) and under IL-2 treatment (right picture: after 9 injections received, 66 MIU in total). (**b**) Representative immunohistochemistry staining of melanoma metastases. Biopsies were taken before and under treatment with IL-2. Scale bar = 100 μm; Insert: scale bar = 50 μm. PD = progressive disease.

Supplementary Table S1: Patient characteristics.

Patient No	Age (Years)	Melanoma Type	ECOG[1]	Stage at Beginning of IL-2 (AJCC[2] 2017)	Biomarkers before Start IL-2			Increase of AEC[5] under IL-2	Number of IL-2 Injections	Total Amount of IL-2 Applied (MIU[6])	Type of Injected Metastases	IL-2-Related Adverse Events	Time Point of Follow up Biopsy Under Il-2 (Months)
					LDH > ULN[3]	S100 > ULN	NLR[4]						
1	69	NM[7]	0	VI (pT3a pN1c M1c)	no	no	7.39	yes	8	24	C[8], SC[9]	Pyrexia, Myalgia	7.0
2	69	NM	0	IIIC (pT4a pN3c cM0)	yes	no	7.39	yes	6	18	C	None	4.5
3	74	ALM[10]	1	IIIC (pT3b pN3c M0)	no	no	0.22	no	15	124	C, SC	Pyrexia, chills, eczema	4.9
4	59	ALM	0	IV (pT3b N1c M1a)	no	no	1.02	yes	19	57	C, SC, N[11]	Dysesthesia, polyneuropathy, cramps, neutropenia	8.2
5	65	ALM	0	IV (pT4b pN3c M1d)	no	no	1.56	no	6	19.5	SC(d)[12]	None	2.6
6	52	mucosal	1	IV (pT4b pN2c M1c)	yes	yes	1.68	no	12	42	C	Nausea, impaired vision, local inflammation	2.4
7	61	NM	0	IIIB (pT2a pN1c M0)	no	yes	1.99	yes	28	252	SC, N	Asthmatic symptoms, pyrexia	1.1
8	66	NM	0	IV (pT3b pN3c M1c)	no	yes	3.76	no	3	21	C, SC	Pyrexia, chills, vomiting, diarrhea	0.5
9	85	SSM[13]	0	IV (pT4b pN1c cM1b)	no	no	2.46	yes	13	102	SC	Pyrexia	1.3
10	54	SSM	0	IV (pT2b pN3c cM1d)	yes	*NA*[14]	2.62	no	2	9	C	none	*NA*
11	36	ALM	0	IIIC (pT3b pN2c cM0)	no	no	1.90	yes	13	58.5	C	Local edema	1.3
12	81	ALM	0	IIIC (pT4b pN2c cM0)	no	no	1.44	no	5	16.5	C	Local inflammation	*NA*
13	80	NM	0	IIIC (pT4b pN1c cM0)	yes	no	5.02	yes	4	6.6	C	None	*NA*
14	88	SSM	0	IIIC (pT4b pN2c cM0)	yes	no	2.30	yes	12	102	C, SC	Pyrexia	*NA*
15	84	NM	1	IV (pT3b pN3c M1a)	*NA*[3]	*NA*	3.77	yes	2	12	C, SC	Local inflammation	*NA*
16	28	CUP[15]	0	IV (T0 Nx M1a)	no	no	1.75	yes	21	96	SC, N	Leucopenia, fatigue, local inflammation	1.7
17	82	Uveal	1	IV (Tx cN0 cM1c)	yes	no	*NA*	*NA*	8	144	C(d)[16], SC(d)	Pyrexia	*NA*
18	69	ALM	0	IV (pT4b pN1c cM1b)	yes	no	6.37	yes	14	180	C, SC, N	Pyrexia, Erysipelas	1.5
19	73	DM[17]	1	IV (pT3b pN3c cM1c)	yes	*NA*	*NA*	no	5	99	C, SC, N	None	*NA*
20	73	ALM	0	IV (pT4b pN2c cM1b)	no	no	*NA*	yes	41	300	C, N	Pyrexia	13.4
21	75	ALM	0	IIIC (pT4 pN3c cM0)	*NA*	*NA*	2.92	no	25	186	C, SC	Pyrexia	*NA*
22	68	NM	0	IIIC (pT3b pN2c cM0)	*NA*	*NA*	3.66	yes	17	150	C	Fatigue, dysesthesia, local inflammation	10.1
23	71	ALM	0	IV (pT4b pN3b cM1c)	*NA*	*NA*	3.10	no	14	84	C(d)	Local itching, flush	*NA*
24	83	NM	0	IIIB (pT3b pN1c cM0)	*NA*	*NA*	2.22	no	5	30	C, SC, N	None	4.1
25	77	ALM	0	IV (pT4b pN3c cM1c)	no	yes	2.58	no	7	27	C	2 x Syncope	*NA*
26	83	ALM	1	IV (pT3b cN2c cM1b)	no	yes	2.37	no	9	93	C, SC	Local pain	*NA*
27	76	SSM	0	IIIC (pT3b pN3c cM0)	*NA*	*NA*	3.03	*NA*	9	40.5	C	Pyrexia, local inflammation, dyspnea, diarrhea	5.1

[1] Eastern Cooperative Oncology Group performance status score. [2] American Joint Committee on Cancer, Melanoma of the Skin Staging, 8th edition. [3] Upper limit normal. [4] Neutrophil-to-lymphocyte ratio. [5] Absolute eosinophil count. [6] Million International Units. [7] Nodular melanoma. [8] Cutaneous (locoregional) [9] Subcutaneous (locoregional). [10] Acral lentiginous melanoma. [11] Nodal (locoregional) [12] Subcutaneous (distant). [13] Superficial spreading melanoma [14] Not available. [15] Cancer of unknown primary. [16] Cutaneous (distant) [17] Desmoplastic melanoma.

Supplementary Table S2. Overall survival times (baseline characteristics).

Condition	Median OS	95% CI[1]	*p* Values
**Sex**			
Female (n = 12)	32	0.0–65.0	0.384
Male (n = 15)	20	12.9–27.1	
**Age over 70**			
No (n = 12)	38	0.0–81.7	0.238
Yes (n = 15)	20	5.2–34.8	
**Loco regional Progression on**			
Adjuvant therapy (n = 9)	32	-*	0.090
Palliative therapy (n = 18)	14	5.2-22.8	
**Active distant metastases at baseline**			
No (n = 13)	NR	-	<0.001
Yes (n = 14)	12	2.8–21.2	
**Baseline LDH[2]**			
Normal (n = 13)	32	4.9–59.2	0.171
Elevated (n = 8)	14	5.7–22.3	
**Baseline S100**			
Normal (n = 14)	32	13.0–51.0	0.325
Elevated (n = 5)	14	0.0–31.2	
**Baseline NLR[3]**			
≤2.5 (n = 12)	32	8.9–55.1	0.533
>2.5 (n = 12)	19	0.0–41.0	
**BRAF-V600-Mutation**			
Wild type (n = 22)	20	18.1–21.9	0.216
Positive (5)	NR	-	

[1] 95% confidence interval of median. [2] Lactate dehydrogenase. [3] Neutrophil-to-lymphocyte ratio.
* No CI due to low event numbers. NR = median not reached. *P*-value indicates statistical significance of the log rank test.

Supplementary Table S3. Overall survival times (values under therapy)

Condition	Median OS	95% CI[1]	*p* Values
**Increase of AEC[2] under IL-2**			
No (n = 12)	13	5.9–20.0	<0.001
Yes (n = 13)	38	-*	
**Loco regional response to IL-2**			
No (n = 8)	8	0.0–17.7	<0.001
Yes (n = 19)	38	23.9–52.1	
**Increase in CD4$^+$ TILs[3]**			
No (n = 5)	19	8.1–29.9	0.004
Yes (n = 11)	NR	-	
**Increase in CD8$^+$ TILs**			
No (n = 4)	12	0.0–28.7	<0.001
Yes (n = 12)	NR	-	
**Increase in PD-1 staining[4]**			
No (n = 6)	19	9.4–28.6	0.194
Yes (n = 10)	38	25.7–50.3	
**Increase in PD-L1 staining[5]**			
No (n = 9)	38	15.4–60.6	0.667
Yes (n = 7)	32	5.1–58.9	

[1] 95% confidence interval of median [2] Absolute eosinophil count. [3] Tumor infiltrating lymphocytes. . [4] Programmed Cell Death Protein 1. [5] Programmed Cell Death 1 Ligand 1. * No CI due to low event numbers. NR = median not reached. *P*-value indicates statistical significance of the log rank test.

Supplementary Table S4. Progression free survival times (Cancer of unknown primary, uveal and mucosal melanoma excluded).

Condition	Median PFS	95% CI[1]	*p* Values
**Active distant metastases at baseline**			
No (*n* = 13)	14	0.0–33.5	0.005
Yes (*n* = 11)	3	0.0–7.0	
**Increase of AEC[2] under IL-2**			
No (*n* = 11)	3	0.0–6.2	<0.001
Yes (*n* = 12)	14	0.0–32.9	
**Loco regional response to IL-2**			
No (*n* = 6)	0	-*	<0.001
Yes (*n* = 18)	12	4.5–19.5	
**Increase in CD4⁺ TILs[3]**			
No (*n* = 5)	7	0.0–22.0	0.224
Yes (*n* = 9)	14	7.5–20.5	
**Increase in CD8⁺ TILs**			
No (*n* = 4)	0	-*	0.010
Yes (*n* = 10)	14	0.0–36.2	
**Increase in PD-1 staining[4]**			
No (*n* = 5)	7	0.0–22.0	0.224
Yes (*n* = 9)	14	7.5–20.5	
**Increase in PD-L1 staining[5]**			
No (*n* = 8)	9	2.1–15.9	0.795
Yes (*n* = 6)	9	3.1–14.9	

[1] 95% confidence interval of median. [2] Absolute eosinophil count. [3] Tumor infiltrating lymphocytes. [4] Programmed Cell Death Protein 1. [5] Programmed Cell Death 1 Ligand 1. * No CI due to low event numbers. *P*-value indicates statistical significance of the log rank test.

Supplementary Table S5. Overall survival times (Cancer of unknown primary, uveal and mucosal melanoma excluded).

Condition	Median OS	95% CI[1]	*p* Values
**Active distant metastases at baseline**			
No ($n = 13$)	NR	-	<0.001
Yes ($n = 11$)	12	6.3–17.7	
**Increase of AEC[2] under IL-2**			
No ($n = 11$)	12	5.6–18.4	<0.001
Yes ($n = 12$)	38	-*	
**Loco regional response to IL-2**			
No ($n = 6$)	8	0.0–20.0	<0.001
Yes ($n = 18$)	38	23.6–52.4	
**Increase in $CD4^+$ TILs[3]**			
No ($n = 5$)	19	8.1–29.9	0.001
Yes ($n = 9$)	NR	-	
**Increase in $CD8^+$ TILs**			
No ($n = 4$)	12	0.0–28.7	<0.001
Yes ($n = 10$)	NR	-	
**Increase in PD-1 staining[4]**			
No ($n = 5$)	19	3.9–34.0	0.052
Yes ($n = 9$)	38	-*	
**Increase in PD-L1 staining[5]**			
No ($n = 8$)	20	17.5–22.5	0.289
Yes ($n = 6$)	32	-*	

[1] 95% confidence interval of median [2] Absolute eosinophil count. [3] Tumor infiltrating lymphocytes. [4] Programmed Cell Death Protein 1. [5] Programmed Cell Death 1 Ligand 1. * No CI due to low event numbers. NR = median not reached. *P*-value indicates statistical significance of the log rank test.
